# Editorial: Vascular malformations: advancements, debates, and consensus

**DOI:** 10.3389/fmed.2025.1680449

**Published:** 2025-08-29

**Authors:** Kaiying Yang, Yi Ji

**Affiliations:** ^1^Department of Dermatology, Zhujiang Hospital, Southern Medical University, Guangzhou, China; ^2^Department of Pediatric Surgery, West China Hospital, Sichuan University, Chengdu, China

**Keywords:** vascular malformation, arteriovenous malformation, venous malformation, lymphatic malformation, therapy

Congenital vascular malformations encompass a broad spectrum of phenotypes, from simple forms such as capillary malformation (CM), venous malformation (VM), arteriovenous malformation (AVM), and lymphatic malformation (LM) to more complex mixed types such as capillary-lymphatic malformation, lymphatic-venous malformation (LVM), and capillary-lymphatic-venous malformation, etc. Although isolated lesions usually have limited clinical impact, syndromic vascular malformations can cause profound functional impairment and may be life-threatening. Given their complex clinical presentations, a deeper understanding of their clinical manifestations, underlying pathogenesis, genetic determinants, and therapeutic approaches is crucial. This Research Topic seeks to synthesize recent advances in these areas and foster consensus to guide future research and clinical management of vascular malformations and related syndromes. Seven articles were included in this Research Topic, comprising four original articles, one review article, one case report, and one brief research report.

## Diverse clinical manifestations of vascular malformation

Lymphedema is characterized by localized tissue swelling resulting from impaired lymphatic drainage due to various underlying causes. It is classified as primary lymphedema (PLE), caused by congenital lymphatic developmental abnormalities, or secondary lymphedema, resulting from lymphatic vessel damage or obstruction, either secondary to diseases such as kaposiform hemangioendothelioma (KHE) (1, 2) or attributable to other etiologies, including infection, trauma, or cancer-related therapies. Zhang et al. compared pediatric PLE and KHE-associated lymphedema, finding that PLE was diagnosed at an older age on average (68.2 vs. 25.0 months), often involved multiple sites, lacked contrast agent accumulation on imaging, and mainly affected the skin and soft tissues rather than the deeper musculoskeletal system. Pham et al. described a rare case of multifocal LVM, initially suspected as a right inguinal hernia on prenatal ultrasound. The male infant presented at 3 months of age with persistent right inguinal and scrotal swelling unresponsive to conservative treatment, and subsequent imaging confirmed the diagnosis, leading to successful therapeutic intervention. In a 10-year series from a German vascular anomaly center, Werba et al. reported that AVMs most frequently involved the hand (32%), typically presented with pain (81%), and showed lesion-specific complications, including necrosis in the hand and growth in the pelvis. These studies illustrate the broad spectrum of clinical presentations associated with vascular malformations, reinforcing the need for careful differential diagnosis.

## Imaging assessment and intraoperative hemorrhage risk stratification in AVM

AVMs are a rare subtype of vascular malformations, accounting for <3% of cases, and are characterized by their fast-flow dynamics resulting from direct connections between high-pressure arteries and low-pressure veins (nidus) (3). Diagnostic imaging plays a pivotal role in both diagnosis and treatment planning of AVMs. In the study by Werba et al., comparative analysis of imaging modalities revealed that digital subtraction angiography (DSA) remains the superior method for treatment planning and nidus evaluation compared with CT or MRI. Among AVMs affecting different anatomical sites, those involving the brain carry particularly severe clinical consequences, given their strong link to hemorrhagic stroke. Brain AVMs account for 25% of hemorrhagic strokes in adults under 50 years of age (4). Consequently, preventing future hemorrhagic episodes is the primary goal of brain AVM management, with microsurgery offering the highest immediate cure rate and the lowest risk of rebleeding, making it a major treatment modality. Shi et al. investigated intraoperative blood loss during brain AVM microsurgery and developed a nomogram to predict major intraoperative blood loss based on five independent factors: (1) clinical manifestations, (2) nidus location, (3) nidus size, (4) deep venous drainage, and (5) the number of draining veins. Collectively, these findings emphasize the value of accurate imaging and tailored surgical risk assessment in improving AVM outcomes.

## Treatment advances in vascular malformation

Traditional treatments for vascular malformations include sclerotherapy, surgical excision, and laser ablation. Surgery is generally reserved due to its potential to impair local tissue function and cause serious complications, while laser ablation is limited by availability and unsuitable for deep or complex lesions. Currently, sclerotherapy remains the first-line therapy owing to its safety and minimal invasiveness. More recently, ultrasound-guided thermal ablation techniques, including radiofrequency ablation (RFA) and microwave ablation (MWA), have gained increasing clinical adoption as alternative minimally invasive treatments. Wang et al. evaluated the safety and efficacy of ultrasound-guided MWA in 39 patients with VMs. After a mean of 1.64 ± 0.87 treatment sessions, all cases achieved a volume reduction exceeding 90%, with the Numeric Rating Scale score decreasing from 5.13 ± 1.65 to 0.53 ± 0.83 (*P* < 0.0001), alongside a marked decrease in lesion volume from 18.34 ± 24.68 ml to 1.35 ± 2.09 ml (*P* = 0.0001). Sporns et al. reported their experience with ultrasound-guided RFA in eight patients with VMs, showing clinical symptom improvement in all cases, with seven achieving complete resolution, one partial relief, and no procedure-related complications. These studies suggest that ultrasound-guided thermal ablation represents a promising, safe, and effective therapeutic option for VMs.

With the identification of specific causative genetic mutations in vascular malformations and an improved understanding of their molecular pathogenesis, several targeted pharmacological therapies have been developed, primarily focusing on the PI3K/AKT/mTOR, RAS/MAPK/ERK, and VEGF signaling pathways, as summarized by Kane and Fernandez-Pineda Sirolimus, an mTOR inhibitor, suppresses lymphangiogenesis and angiogenesis through inhibition of the PI3K/AKT/mTOR pathway and has demonstrated substantial therapeutic potential in vascular anomalies (5). It is now established as a first-line therapy for vascular tumors such as KHE (6) and has shown efficacy in various vascular malformations, including common LM and kaposiform lymphangiomatosis (3, 7). Other targeted agents include alpelisib, a selective PIK3CA inhibitor that has achieved favorable clinical outcomes in patients with GLOVES syndrome and PIK3CA-related overgrowth spectrum (8, 9), likely by reducing phosphorylated AKT and inhibiting mTORC1 activation. Additionally, the MEK inhibitor trametinib has been reported to benefit patients harboring RAS/MAPK/ERK pathway mutations, particularly those with AVMs (10). Collectively, these advances underscore the growing promise of molecularly targeted therapies as precision medicine strategies for the management of vascular malformations.

## Conclusion

The articles in this Research Topic emphasize the diverse clinical manifestations of vascular malformations, advancements in imaging and minimally invasive treatments, and the promise of targeted therapies. Together, they underscore the shift toward precision medicine in vascular malformation management. However, challenges remain in fully elucidating underlying mechanisms and long-term treatment outcomes. Future investigations should focus on integrating molecular insights with clinical phenotypes to guide optimized, individualized management strategies.
